# Incidence, Prevalence, and Risk Factors of Hemiplegic Shoulder Pain: A Systematic Review

**DOI:** 10.3390/ijerph17144962

**Published:** 2020-07-09

**Authors:** Shahnawaz Anwer, Ahmad Alghadir

**Affiliations:** 1 Department of Rehabilitation Sciences, College of Applied Medical Sciences, King Saud University, Riyadh 11433, Saudi Arabia; aalghadir@hotmail.com; 2 Department of Building and Real Estate, Hong Kong Polytechnic University, Kowloon, Hong Kong

**Keywords:** stroke, pain, hemiplegic shoulder pain, incidence, prevalence, risk factors

## Abstract

The current systematic review aimed to investigate the incidence, prevalence, and risk factors causing hemiplegic shoulder pain (HSP) after stroke. Two independent authors screened titles and abstracts for the eligibility of the included studies in the electronic databases PubMed and Web of Science. Studies which reported the incidence, prevalence, and risk factors of HSP following stroke were included. The included studies were assessed using the Newcastle–Ottawa Scale for evaluating the quality of nonrandomized studies in meta-analyses. Eighteen studies were included in the final synthesis. In all studies, the number of patients ranged between 58 and 608, with the mean age ranging from 58.7 to 76 years. Seven included studies were rated as “good “quality, while one study rated “fair” and 10 studies rated “poor” quality. Eight studies reported incidence rate while 11 studies reported the prevalence of HSP following a stroke. The incidence of HSP was ranging from 10 to 22% in the metanalysis of the included studies. The prevalence of HSP was ranging from 22 to 47% in the metanalysis of the included studies. The most significant predictors of HSP were age, female gender, increased tone, sensory impairment, left-sided hemiparesis, hemorrhagic stroke, hemispatial neglect, positive past medical history, and poor National Institutes of Health Stroke Scale score. The incidence and prevalence of HSP after stroke vary considerably due to various factors. Knowledge of predictors is important to minimize the risk of developing HSP following a stroke.

## 1. Introduction

Hemiplegic shoulder pain (HSP) is a common and disabling complication following a stroke, and it may affect the quality of life [1]. It often occurs following two to three months of stroke [2,3]. Consequently, HSP may result in withdrawal from rehabilitation programs, longer hospital stays, reduced limb movement, and impaired quality of life [1]. Numerous causes have been implicated in developing HSP in stroke. This includes muscle flaccidity around the shoulder joint, shoulder subluxation, shoulder-hand syndrome, increased muscle tone, impingement syndrome, frozen shoulder, brachial plexus injury, and the thalamic syndrome [4,5].

There is an inconsistency in reporting incidence and prevalence of HSP following stroke. Some studies reported that the incidence of HSP ranges from 16% to 84% [6,7], while others reported a range of 65% to 70% [8,9]. In other studies, the incidence rate was ranging from 24% to 64% in those stroke populations who are admitted to an inpatient rehabilitation unit [10,11,12,13] and it varied from 9% to 40% in those who are not admitted [14,15,16].

A reduced arm motor function at the time of stroke or over a period during rehabilitation was found to be one of the risk factors for developing HSP following stroke [6,10]. Additionally, HSP often occurs and tends to be more severe among people with left-sided hemiplegia [15,17,18]. Reduced shoulder motion in the affected side of persons with HSP is presented in the initial weeks following stroke [19] and it gradually worsens after one month [19,20,21]. Other factors probably causing HSP can include shoulder subluxation [22] or rotator cuff injury [23], and those which are related to the neurological problem such as impaired sensation, hemispatial neglect, spasticity, and flaccid paralysis [24,25].

Despite many observational and interventional studies, incidence, prevalence, and the clinical presentations of HSP differ and the exact underlying factors causing HSP are unknown, resulting lack of knowledge in designing effective strategies to prevent and treat HSP. Currently, there were no studies systematically explored the incidence, prevalence, and the risk factors causing shoulder pain in patients with stroke. Therefore, the current systematic review aimed to investigate the incidence, prevalence, and risk factors causing HSP after stroke.

## 2. Materials and Methods

This systematic review followed the guideline for the Meta-analysis Of Observational Studies in Epidemiology (MOOSE) [26]. This systematic review was prospectively registered in PROSPERO (CRD42017077594) and available at http://www.crd.york.ac.uk/PROSPERO/display_record.php?ID=CRD42017077594.

### 2.1. Search Strategy

The electronic databases PubMed, Web of Science, and Scopus were searched until November 19, 2019. Additionally, potential articles were searched manually from the reference list given in each article. The literature search in PubMed was conducted using the following keywords: (“Stroke” OR “Hemiplegia”) AND (“Shoulder” OR “Arm” OR “Shoulder joint”) AND (“Shoulder pain” OR “Pain”) AND (“Prevalence” OR “Incidence”) [Table 1].

### 2.2. Eligibility Criteria

The current review included all the published literature that qualified the following criteria: studies included adults over 18 years of age with a history of stroke for more than one month; the outcomes of included studies should be pain in and around shoulder excluding ribs and the neck pain; all cross-sectional and longitudinal studies to assess the natural course of events after stroke and; studies describing potential risk factors such as demographic factors or impairments related to post-stroke shoulder pain that was evaluated after the incidence of stroke.

Studies were excluded if they were not published in English. Additionally, case reports and case series were also excluded as these types of studies might have a high potential bias. Furthermore, if the cause of shoulder pain was not secondary to stroke, those studies were also excluded (for instance, shoulder pain due to neck pathology).

### 2.3. Study Selection

Two independent authors screened titles and abstract for the eligibility of the included studies. Studies, which reported the incidence, prevalence, and risk factors of HPS following stroke were included. Any disagreements between two reviewers in the study selection were discussed and resolved by consensus between them.

### 2.4. Data Extraction and Assessment of the Risk of Bias

Two independent authors (SA and AA) completed the data extraction and assessment of the risk of bias, using structured formats. The important data extraction included the following items: author’s name, setting, country of origin, sample size, target population, time since stroke (months), the average age of patients, outcome measurement, and study design. Quality assessment and risk of bias in the selected studies were appraised using the Newcastle-Ottawa Scale for evaluating the quality of nonrandomized studies in meta-analyses [27,28]. This evaluation tool has considered three factors (e.g., selection of exposed and non-exposed cohort, comparability of cohorts based on the design or analysis, and outcome based on reliability and validity of the scale, adequate follow-up and dropout rate) to appraise the quality of each included study [27,28]. The quality of each study was rated as good, fair, and poor by assigning stars in each domain as per given the guidelines of the Newcastle-Ottawa Scale [27,28]. A “good” quality score was given if the included study received 3 or 4 stars in selection domain,1 or 2 stars in comparability domain, and 2 or 3 stars in outcome domain. A “fair” quality score was given if the included study received 2 stars in selection domain,1 or 2 stars in comparability domain, and 2 or 3 stars in outcome domain. A “poor” quality score indicated 0 or 1 star(s) in selection domain, or 0 stars in comparability domain, or 0 or 1 star(s) in outcome domain (Table 2). Any disagreements between two reviewers were discussed and resolved by consensus between them.

### 2.5. Summary Statistics and Synthesis of Results

Incidence and prevalence of HSP were reported from the included studies. Prevalence of HSP in the defined group was also reported if the information is available in the included studies. Most significant predictors of HSP after stroke were determined from the included studies. The Comprehensive Meta-Analysis software was used to conduct a meta-analysis. Meta-analysis was conducted for the prevalence, incidence, and risk factors of shoulder pain after stroke if at least two or more included studies with the adequate data for the analysis were available. The event rates for the prevalence and incidence estimates and the odd ratios (ORs) for the risk factors of shoulder pain after stroke were calculated. The effect size with 95% confidence intervals (CIs) for the prevalence, incidence, and risk factors of shoulder pain after stroke were calculated.

## 3. Results

### 3.1. Study Selection

**Figure 1:**
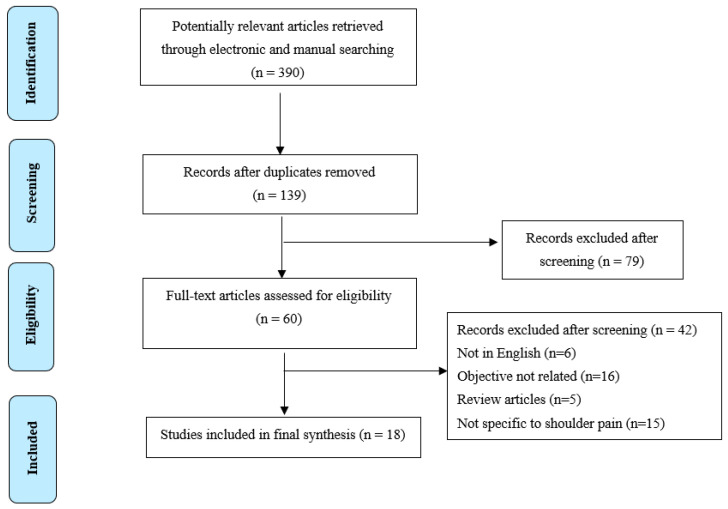
Study selection process and results of the literature search (PRISMA flow diagram).

Based on the abstract and title search, initially, 390 articles were identified. After excluding duplicates (*N* = 251) and screening of abstracts, a total of 330 studies were not relevant to the current review, therefore excluded. Sixty full-text articles were included in the final screening, of which 42 articles failed to match the inclusion criteria, and were hence excluded. Henceforth, a total of 18 studies included in the final synthesis [3,6,9,11,14,16,28,29,30,31,32,33,34,35,36,37,38,39]. Figure 1 shows the details of the study selection process and results of the literature search [26].

### 3.2. Characteristics of Included Studies

Table 3 present the characteristics of included studies. Among 18 included studies [3,6,9,11,14,16,29,30,31,32,33,34,35,36,37,38,39,40], sixteen were categorized as a prospective observational study [3,6,9,14,16,29,30,31,32,33,34,35,37,38,39,40], while others as a retrospective observational study [11,36]. Studies originated from the UK [14,16,29,30], Turkey [9,39], Sweden [6,34], Thailand [31,35], Australia [3,11], Denmark [32,33], Taiwan [36], Korea [37,38], and Italy [40]. In all studies, the number of patients ranged between 58 and 608 with the mean age ranging from 58.7 to 76 years. Four included studies have defined participants as first-ever stroke patients [6,34,36,37]. Most of the other studies have defined participants as patients with stroke [3,11,14,31,32,33,35,38,39,40], while two studies [16,29] have defined participants as patients with a diagnosis of acute stroke. In all studies, the time since incidence of a stroke at recruitment was within one week of symptom onset up to 30 months after stroke. Seven include studies used a visual analog scale (VAS) [3,6,14,16,29,30,34], two studies used a numerical rating scale (NRS) [32,37], one study used a Neuropathic Pain Symptom Inventory [40], one study used the University of Alabama’s Pain Behaviors Scale [38], one study used medical records [36], and one study used an interview method [33], while others used physical examination to assess shoulder pain after stroke [9,11,31,35,39].

### 3.3. Methodological Quality

Table 2 presented the results of the quality assessment using the Newcastle–Ottawa Scale for evaluating the quality of nonrandomized studies in meta-analyses [27,28]. Seven included studies [3,6,14,16,34,35,37] were rated as “good “quality, while one study [39] rated “fair” and 10 studies [9,11,29,30,31,32,33,36,38,40] rated “poor” quality. Five included studies [14,30,33,36,40] did not have any non-exposed control group. More than half of the included studies [3,9,11,31,33,35,36,38,39,40] had reported the presence of outcome at the start of the study. Eight included studies [9,11,29,31,32,36,38,40] reported less than 6-month of follow-up. Nine included studies [9,11,29,31,32,36,38,39,40] did not provide information about the dropouts.

### 3.4. Incidence and Prevalence of HSP after Stroke

The details of the incidence and prevalence of HSP after stroke is given in Table 4. The overall incidence of HSP was ranging from 1.6 to 40% in the included studies. The overall prevalence of HSP was ranging from 9.41 to 91.9% in the included studies. The incidence of HSP after a stroke at admission was reported in two studies [3,14]. Incidence at admission ranged from 9 to 10%. Three studies reported on the prevalence of HSP after a stroke at admission [9,11,39], ranging from 23 to 63.5%. The incidence of HSP after a stroke at 6 months was reported in three studies [14,16,33], ranging from 15 to 40%. Only one study reported the 6-month prevalence (42%) of HSP after a stroke [30]. Only one study reported the 12-month incidence (21%) of HSP after stroke [3]. Two studies reported the 12-month prevalence of HSP after stroke [30,35], ranging from 8.5 to 47%. Only one study reported the two-year incidence (15.1%) of HSP after a stroke [32].

### 3.5. Risk Factors of HSP after Stroke

Table 5 presented the risk factors of HSP used in the included studies. A variety of risk factors was assessed in the included studies [3,6,9,11,14,16,29,31,32,34,37,39]. The most significant risk factors of HSP were age, arm weakness, sensory impairment, abnormal shoulder joint examination, average depression score, functional status, self-perceived ill health, subluxation, pathogenesis, left-sided hemiparesis, and prior history of shoulder pain. Six studies did not assess the risk factors of HSP after stroke [30,33,35,36,38,40].

### 3.6. Meta-Analysis

**Figure 2:**
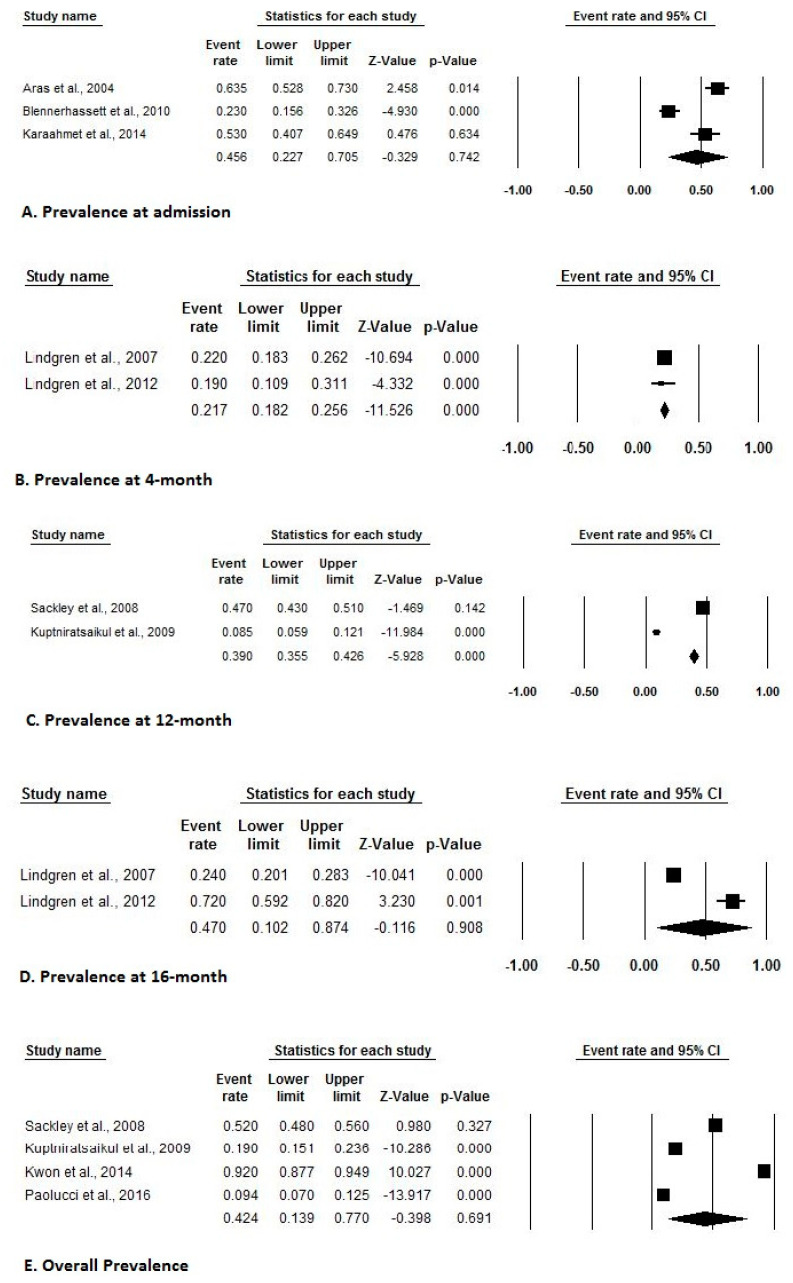
Prevalence of shoulder pain after stroke ((**A**). Prevalence at admission, (**B**). Prevalence at 4-month, (**C**). Prevalence at 12-month, (**D**). Prevalence at 16-month, (**E**). Overall prevalence).

**Figure 3:**
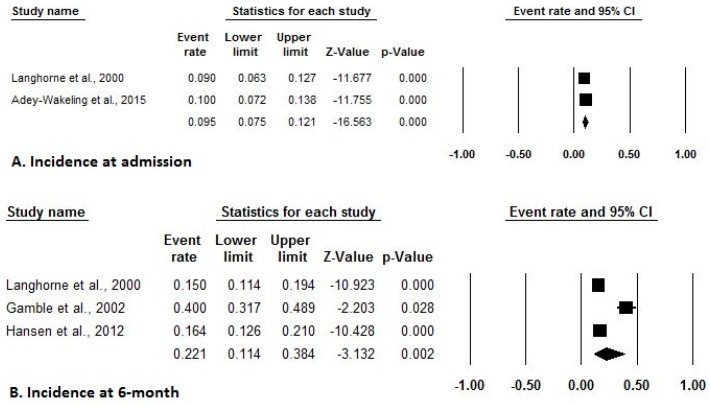
Incidence of shoulder pain after stroke ((**A**). Incidence at admission, (**B**). Incidence at 6-month).

**Figure 4:**
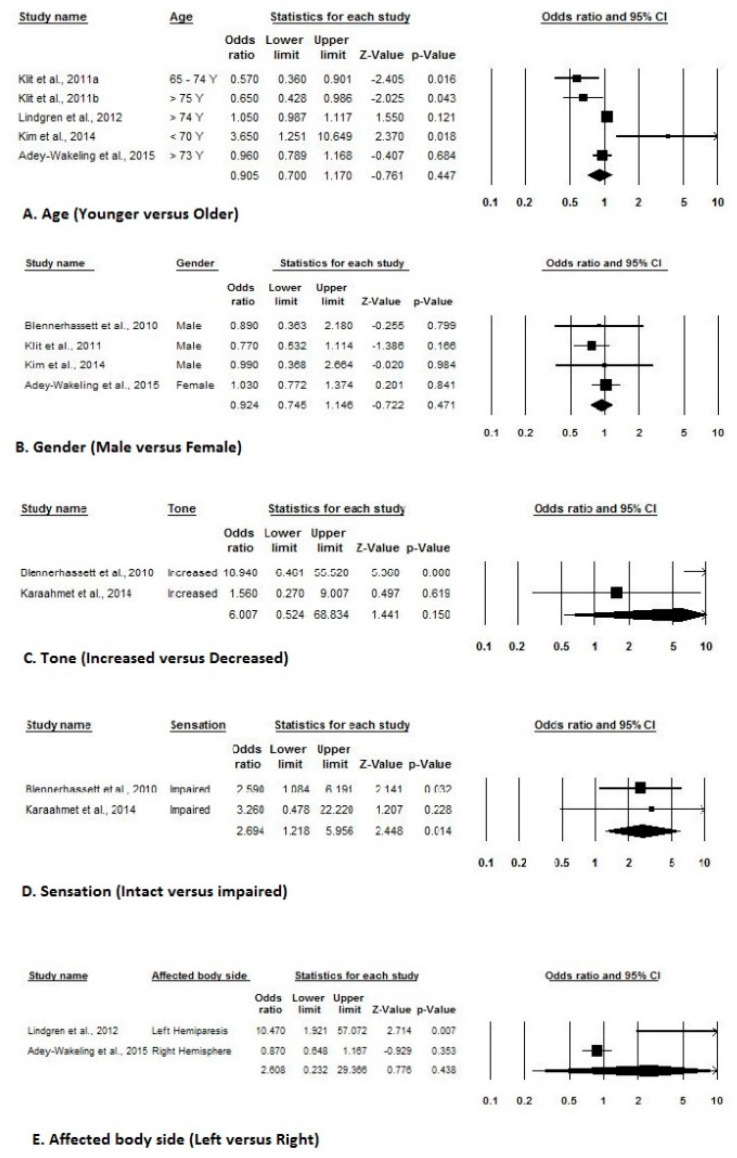
Demographic risk factors for shoulder pain after stroke ((**A**). Age, (**B**). Gender, (**C**). Table 5. Clinical risk factors for shoulder pain after stroke ((**A**). Types of stroke, (**B**). Hemispatial neglect, (**C**). Past medical history, (**D**). National Institutes of Health Stroke Scale-NIHSS Score).

**Figure 5:**
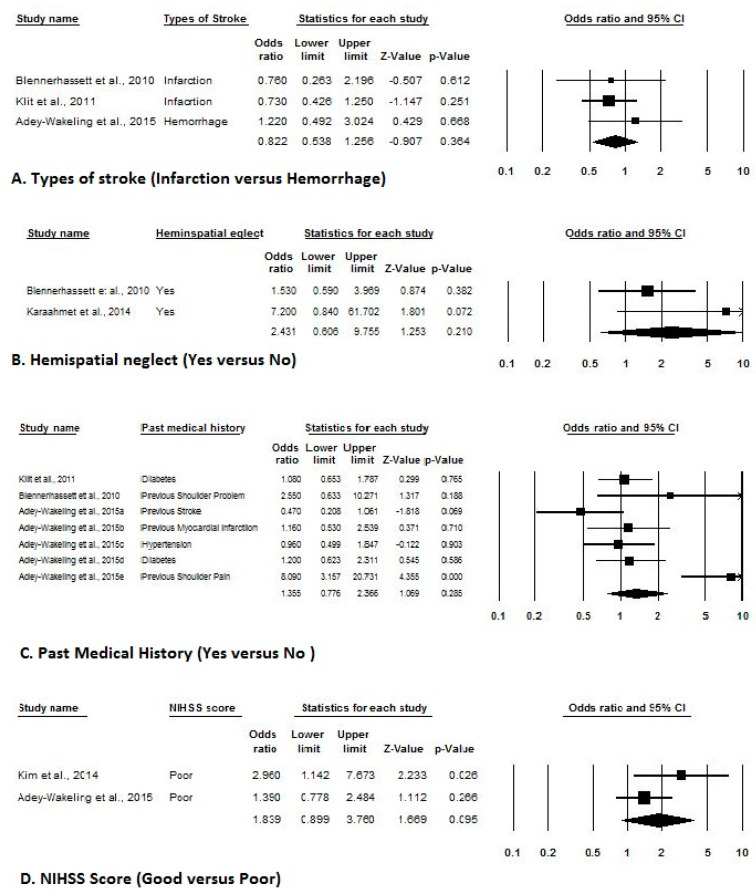
Clinical risk factors for shoulder pain after stroke ((**A**). Types of stroke, (**B**). Hemispatial neglect, (**C**). Past medical history, (**D**). National Institutes of Health Stroke Scale-NIHSS Score).

Of the 10 included studies with adequate prevalence data, three estimated event rates for the prevalence at admission, two estimated event rates for the prevalence at 4-month, 12-month, and 16-month, while four estimated event rates for an overall prevalence of shoulder pain after stroke. Figure 2 indicates a forest plot of prevalence rates with effect sizes and 95% CIs. Of the five included studies with adequate incidence data, two estimated event rates of the incidence at admission, while three estimated event rates of incidence at 6-month of shoulder pain after stroke. Figure 3 indicates a forest plot of incidence rates with effect sizes and 95% CIs. Of the eight included studies with adequate risk estimate data, three studies estimated ORs stratified by gender, two stratified by tone, sensation, affected body side, hemispatial neglect, and National Institutes of Health Stroke Scale Score, while three stratified based on type of strokes and seven stratified based on past medical history. Figure 4 and Figure 5 indicate a forest plot of risk factors with effect sizes and 95% CIs.

## 4. Discussion

Many reviews on HSP have been published in recent decades [41,42,43,44,45,46]. However, these reviews focus on the cause and treatment of HSP after stroke. As per our knowledge, this is the first systematic review, presenting the results of 18 studies on the incidence and prevalence of HSP after stroke. Additionally, the present review assessed the risk factors causing HSP after stroke. In the current review, a total of 5086 patients with a mean age ranging from 58.7 to 76 years were included. Seven included studies were rated as “good” quality, while one study rated “fair”, and 10 studies rated “poor” quality. Eleven studies reported the prevalence, while eight studies reported the incidence rate of HSP following a stroke. The incidence of HSP was ranging from 10 to 22% in the metanalysis of the included studies. The prevalence of HSP was ranging from 22 to 47% in the metanalysis of the included studies.

Previous studies reported the prevalence of shoulder pain after stroke between 21% and 84% [6,12,14,16,30], while others reported 5% and 84% [47,48]. The wide variation in the prevalence of HSP reflects the lack of proper definition or an inconsistency in the quality of care of these patients among diverse populations [49,50]. Incidence at admission was approximately 10% in the present review. The incidence of HSP after a stroke at 6 months was 22% in the present review. Wanklyn et al. [51] reported that about 63% of the patients developed HSP in the first six months after stroke. Other studies reported that the HSP usually develops within 2–3 months following the stroke onset [2,6,16]. In the current review, 12 months prevalence of HSP after a stroke was 39%. A previous study reported the prevalence of HSP about 32% within the 12 months after a stroke [52]. Another study reported the prevalence of HSP about 34% at 12 months after a stroke [36].

In the current review, the most significant identified predictors of HSP were age (younger than 70 years), female gender, increased tone, sensory impairment, left-sided hemiparesis, hemorrhagic stroke, hemispatial neglect, positive past medical history, and poor National Institutes of Health Stroke Scale score. In the previous study, age was not directly linked to the development of HSP, but older people could have preexisting problems that affect shoulder pain [53]. Another study reported various risk factors, including impaired motor control, reduced proprioception, sensory impairment, spasticity of the elbow flexor muscles, limited range of motion (ROM) of the shoulder joint, and type 2 diabetes mellitus [54]. Barlak et al. [13] reported a significant correlation between HSP and complex regional pain syndrome and adhesive capsulitis, but there was no correlation found between the HSP and the grade of subluxation, impingement syndrome or spasticity. Similarly, other studies also reported many risk factors for shoulder pain, and the severity of motor impairment is one of the most important risk factors identified [6,15,16,40,51,54].

During the recovery from stroke, muscle spasticity of the upper extremities is thought to cause shoulder subluxation and limited ROM, resulting in the development of shoulder pain [55]. Another important cause of HSP is frozen shoulder (Adhesive capsulitis), which is indicated by a limited shoulder ROM, with a capsular type of restriction [56,57,58]. However, other factors could also cause a reduced shoulder ROM in stroke patients without frozen shoulder. Mao et al. [59] identified a prolonged, shortened position was one of the causes of developing soft tissues contracture around the shoulder such as muscles, tendons, and ligaments. The current review was not able to identify a single pathology causing shoulder pain in patients with stroke. This area of research should be explored in the future investigations.

The major strength of this review was the inclusion of a metanalysis, which identified many risk factors causing shoulder pain after stroke. Additionally, most of the included studies were categorized as a prospective observational study, which further enhances the validity of the results. The present study had some potential limitations as well. Some of the included studies did not use a valid and reliable outcome measure to assess symptoms of shoulder pain, which could affect the validity of the results. Additionally, most of the included studies did not report the exact cause of shoulder pain following stroke. Furthermore, variations in the participants’ characteristics in the included studies prevent to make any causal relationships between shoulder pain and predictor measures.

## 5. Conclusions

The incidence and prevalence of shoulder pain after stroke vary considerably due to various factors. Knowledge of predictors is important to minimize the risk of developing shoulder pain following a stroke.

## Figures and Tables

**Table 1:** Search Strategy.

	PubMed	Web of Science	Scopus
Date	19-Nov-2019	19-Nov-2019	19-Nov-2019
“Stroke” OR “Hemiplegia”	291,950	296,060	395,489
“Shoulder” OR “Arm” OR “Shoulder joint”	213,412	228,005	450,679
“Shoulder pain” OR “Pain”	678,816	538,019	1,038,105
“Prevalence” OR “Incidence”	1,323,666	1,288,045	1,882,741
Combined search	94	115	181
Total minus duplicates	139		

**Table 2:** Results of the quality assessment using the Newcastle-Ottawa Quality Assessment Scale criteria.

Studies	Selection				Comparability	Outcome			Quality Score †
	Representativeness of the exposed cohort	Selection of the non-exposed cohort	Ascertainment of exposure	Outcome of Interest Was Not Present at Start of Study	Comparability of cohorts based on the design or analysis	Assessment of outcome	Follow-Up Long Enough for Outcome to Occur (Median Duration of Follow-Up ≥ 6 Months)	Adequacy of follow up of cohorts	
Langhorne et al. (2000) [14]	Three major hospitals in the West of Scotland were participated. Two hospitals provided acute stroke patient care and one hospital provide acute stroke rehabilitation care after one-week discharge. ★	No non-exposed cohort	Weekly assessments in hospitals by 3 research nurses ★	Yes ★	Complication subdivided by their baseline level of dependency and compared using Chi-square test. ★	Questionnaire based structured interview ★	Yes ★	100% participated at the 6-month follow-up, 99% at the 18-month follow-up, and 93% at the 30-month. ★	Good
Gamble et al. (2000) [29]	Consecutive cohort of stroke patients admitted to a single hospital.	Yes ★	Patients underwent interview about shoulder pain ★	Yes ★	Age, sex, level of anxiety, disability score, or moderate to severe motor weakness were adjusted in chi-square test. ★	Questionnaire based structured interview ★	No	Not reported	Poor
Gamble et al. (2002) [16]	Consecutive cohort of stroke patients admitted to a single hospital.	Yes ★	Patients underwent interview about shoulder pain ★	Yes ★	Age, sex, depression and anxiety scores, and functional scores were adjusted in chi-square test. ★	Questionnaire based structured interview ★	Yes ★	97% participated at the 6-month follow-up. ★	Good
Aras et al. (2004) [9]	Consecutive cohort of stroke patients admitted to a single hospital.	Yes ★	Patients underwent interview about shoulder pain ★	No	The presence of spasticity, thalamic pain, neglect, and comorbidities were compared between groups with and without shoulder pain. ★	Questionnaire based structured interview ★	No	No statement	Poor
Lindgren et al. (2007) [6]	Participants were representative of the Lund Stroke Register which covers the population of Lund-Orup, including 8 municipalities representing the local geographical area of Lund University Hospital. ★	Yes ★	Patients underwent interview about shoulder pain ★	Yes ★	Univariate analyses were used to compare arm motor function, disability, self-perceived health, subluxation, and sensory disturbance, between patients with and without shoulder pain. ★	Questionnaire based structured interview ★	Yes ★	79% participated at the 4-month follow-up, and 73% at the 12-month follow-up. ★	Good
Sackley et al. (2008) [30]	Potential participants were representative of the Nottingham Stroke Register, which includes all stroke admissions to Nottingham City Hospital and Queens Medical Centre, Nottingham, UK. ★	No non-exposed cohort	Patients underwent interview about shoulder pain ★	Yes ★	confounders were not reported.	Questionnaire based structured interview ★	Yes ★	84% participated at the 3-month follow-up, 61% at the 6-month follow-up, and 50% at the 12-month follow-up. ★	Poor
Kuptniratsaikul et al. (2009) [31]	Participants were representative of Thai Stroke Rehabilitation Registry, which maintain the record of patients with stroke who underwent rehabilitation in Thailand. ★	Yes ★	Patients underwent interview about shoulder pain ★	No	Confounders were compared using the multivariate analysis. ★	Questionnaire based structured interview ★	No	No statement	Poor
Blennerhassett et al. (2010) [11]	The 94 retrospective histories of patients admitted for inpatient rehabilitation were audited which represented 63% of stroke patients in a 3-year period.	Yes ★	Medical report	No	Confounders were compared using the logistic regression analysis. ★	Retrospective data	No	No statement	Poor
Klit et al. (2011) [32]	Participants were representative of National Indicator Project database which records all hospitalized acute stroke patients in Denmark. ★	Yes ★	Patients reported shoulder pain	Yes ★	Confounders were compared using the multiple logistic regression analysis. ★	Questionnaire-based survey	No	No statement	Poor
Hansen et al. (2012) [33]	Consecutive cohort of stroke patients admitted to a single hospital.	No	Patients underwent a structured interview. ★	No	Age and gender were adjusted in chi-square test. ★	Questionnaire based structured interview ★	Yes ★	97% participated at the 3-month follow-up, and 92% at the 6-month follow-up. ★	Poor
Lindgren et al. (2012) [34]	Participants were representative of the Lund Stroke Register which covers the population of Lund-Orup, including 8 municipalities representing the local geographical area of Lund University Hospital. ★	Yes ★	Patients underwent interview about shoulder pain ★	Yes ★	Univariate analyses were used to compare age, pain frequency, affected side, motor function, and passive range of abduction between patients with and without shoulder pain. ★	Questionnaire based structured interview ★	Yes ★	79% participated at the 4-month follow-up, and 73% at the 12-month follow-up. ★	Good
Kuptniratsaikul et al. (2013) [35]	Participants were representative of Thai Stroke Rehabilitation Registry, which maintain the record of patients with stroke who underwent rehabilitation in Thailand. ★	Yes ★	Patients underwent interview about shoulder pain ★	No	Confounders were compared using the multivariate analysis. ★	Questionnaire based structured interview ★	Yes ★	65% participated at the 12-month follow-up. ★	Good
Chen et al. (2014) [36]	The medical records of patients consecutively admitted to a single hospital.	No	Retrospective data	No	Confounders were compared using the Chi-square test. ★	Retrospective medical record data	No	No statement	Poor
Kim et al. (2014) [37]	Consecutive cohort of stroke patients admitted to a single hospital.	Yes ★	Patients underwent interview about shoulder pain ★	Yes ★	Age, gender and significant variables from the univariate analysis were included in the final multivariate logistic regression model. ★	Questionnaire based structured interview ★	Yes ★	78% participated at the 3-month follow-up, and 62% at the 6-month follow-up. ★	Good
Kwon et al. (2014) [38]	Participants were representative of eight rehabilitation units situated in three different large local catchment area in the Republic of Korea. ★	Yes ★	Patients underwent interview about shoulder pain ★	No	Age, sex, the Motricity Index of the upper and lower limbs, and ambulatory types were included in the multivariate logistic regression model. ★	Questionnaire based structured interview ★	No	No statement	Poor
Karaahmet et al. (2014) [39]	Consecutive cohort of stroke patients admitted to a single physical medicine and rehabilitation clinic.	Yes ★	Patients underwent interview about shoulder pain ★	No	Disease duration, neglect, sensory disturbance, spasticity, immobilization, late rehabilitation, and motor function were included in backward stepwise multinomial logistic regression analysis. ★	Questionnaire based structured interview ★	Yes ★	No statement	Fair
Adey-Wakeling et al. (2015) [3]	A population-based stroke incidence study conducted in a specific region of the western suburbs of Adelaide, South Australia. ★	Yes ★	Patients underwent interview about shoulder pain ★	No	logistic regression models were used to analyses different confounders. ★	Questionnaire based structured interview ★	Yes ★	78% participated at the 4-month follow-up, and 75% at the 12-month follow-up. ★	Good
Paolucci et al. (2016) [40]	Consecutive cohort of stroke patients admitted to at eight Italian hospitals. ★	No	Patients underwent interview about shoulder pain ★	No	Age, gender, type of stroke, and severity of stroke were included in regression analysis. ★	Questionnaire based structured interview ★	No	No statement	Poor

**†** Good quality: 3 or 4 stars (★) in selection domain AND 1 or 2 stars in comparability domain AND 2 or 3 stars in outcome domain; Fair quality: 2 stars in selection domain AND 1 or 2 stars in comparability domain AND 2 or 3 stars in outcome/exposure domain; Poor quality: 0 or 1 star in selection domain OR 0 stars in comparability domain OR 0 or 1 stars in outcome/exposure domain.

**Table 3:** Study characteristics.

	Setting/Country	Sample Size	Targeted Population	Time since Stroke (Months) at Recruitment	Average Age at Recruitment	Outcome Measurement	Design
Langhorne et al. (2000) [14]	Multicenter hospital-based study/Scotland, UK.	*N* = 311	People with stroke, Hemiplegia	up to 30 months after stroke	76 years (interquartile range 70 to 82 years)	VAS	A prospective study
Gamble et al. (2000) [29]	Hospital-based study/UK	*N* = 123	Patients with a diagnosis of acute stroke	up to 6 months	70.6 years	VAS	A prospective study
Gamble et al. (2002) [16]	Hospital-based study/UK	*N* = 123	Patients with a diagnosis of acute stroke	up to 6 months	70.6 years (range 29–93)	VAS	A prospective study
Aras et al. (2004) [9]	Hospital-based study/Turkey	*N* = 85	Patients with hemiplegia	64.8 days from the onset	58.7 years	Physical examination	A prospective study
Lindgren et al. (2007) [6]	Population-Based Study/Sweden	*N* = 416	First-ever stroke patients	up to 16 months	73.1 years (range 17–102 years)	VAS	A prospective study
Sackley et al. (2008) [30]	Hospital-based study/UK	*N* = 600	3-months post stroke	3-months from the onset up to 12-months	76 years (range 31–98 years)	VAS	A prospective study
Kuptniratsaikul et al. (2009) [31]	Multicenter hospital-based study/Thailand	*N* = 327	Patients with stroke	more than two months	62.2 years (SD 12.1)	Physical examination	A prospective study
Blennerhassett et al. (2010) [11]	Hospital-based data/Australia	*N* = 94	Patients with stroke	More than 2 months	59 years (range 17–80 years)	Physical examination	Retrospective observational study
Klit et al. (2011) [32]	Population-based study/Denmark	*N* = 608 (stroke patients), 519 (reference subjects)	Patients with stroke	Median days from stroke 794.5 (range 588–1099)	Median age, 72.6 years	NRS	A prospective study
Hansen et al. (2012) [33]	Hospital-based study/Denmark	*N* = 299	Patients with stroke	up to 6 months post stroke	65.6 years (24–92 years)	Interview	A prospective study
Lindgren et al. (2012) [34]	Hospital-based study/Sweden	*N* = 58	First-ever stroke patients	up to 16 months	71 years	VAS	A prospective study
Kuptniratsaikul et al. (2013) [35]	Multicenter hospital-based study/Thailand	*N* = 327	Patients with stroke	12 months of onset	62.1 years (SD 12.5 years)	Physical examination	A prospective study
Chen et al. (2014) [36]	Hospital-based study/Taiwan	*N* = 568	First-time stroke patients	Not reported	65.7 years (SD 13.3 years)	Medical records	A retrospective longitudinal cohort study
Kim et al. (2014) [37]	Hospital-based study/Korea	*N* = 94	Patients with first-ever unilateral stroke lesion	up to 6 months post-stroke	65.6 years	NRS	A prospective study
Kwon et al. (2014) [38]	Hospital-based study/Korea	*N* = 229	Patients with stroke	More than 2 months	59.0 years (SD 12.4)	University of Alabama’s Pain Behaviors Scale	A prospective study
Karaahmet et al. (2014) [39]	Hospital-based study/Turkey	*N* = 63	Patients with stroke	More than 2 months	61 years (range, 39–85 years)	Physical examination	A prospective study
Adey-Wakeling et al. (2015) [3]	Population-Based Study/Australia	*N* = 318	Patients with stroke	Average 8.7 days post onset up to 12 years	72.5 years	VAS	A prospective study
Paolucci et al. (2016) [40]	Hospital-based multicenter study/Italy	*N* = 443	Patients with stroke	more than 90 days onset of stroke	67.1 years	Neuropathic Pain Symptom Inventory	A prospective study

VAS: Visual analogue scale; NRS: Numerical rating scale.

**Table 4:** Incidence and prevalence of shoulder pain after stroke in the included studies.

Study	Incidence [Proportion (95% CI)]	Prevalence [Proportion (95% CI)]	Prevalence in Defined Group
L	Incidence at admission: 9% (6–12%)	Weekly point prevalence: 6% (5–7%)	
	Discharge to 6-month incidence: 15% (9–21%)		
	6-months to 18-months incidence: 11% (6–16%)		
	18-months to 30-months incidence: 12% (6–17%)		
Gamble et al. (2000) [29]	Incidence at 2-week: 25%		
Gamble et al. (2002) [16]	Incidence at 6-months: 40%		
Aras et al. (2004) [9]		Prevalence at admission: 63.5%	
Lindgren et al. (2007) [6]		Prevalence at 4-months: 22% Prevalence at 16-months: 24%	Functional status independence: 37%
			Moderate dependence: 31%
			Major dependence: 32%
			Self-perceived ill health: 23%
			Arm motor function
			No function: 27%
			Reduced function: 56%
			Normal function: 17%
			Sensory disturbance for light touch: 31%
			Shoulder Subluxation: 41%
Sackley et al. (2008) [30]		Overall prevalence: 52%	
		Prevalence at 3-months: 36%	
		Prevalence at 6-months: 42%	
		Prevalence at 12-months: 47%	
Kuptniratsaikul et al. (2009) [31]		Overall prevalence: 19%	Hemorrhagic stroke, prevalence of shoulder pain: 26.1%
			Infarction stroke, prevalence of shoulder pain: 16.2%
Blennerhassett et al. (2010) [11]	Incidence during inpatient: 11.7%	Prevalence at admission: 23%	
		Prevalence during inpatient: 35%	
Klit et al. (2011) [32]	Two-year incidence: 15.1%		
Hansen et al. (2012) [33]	Incident at onset: 1.5%		Shoulder pain in stroke-affected side at onset: 1.1%
	Incident at 3-months: 13.1%		at 3-months: 10.2%
	Incident at 6-months: 16.4%		at 6-months:12.0%
Lindgren et al. (2012) [34]		Prevalence at 4 and 16-months: 19% and 72%, respectively	
Kuptniratsaikul et al. (2013) [35]		Prevalence at 12-months: 8.5%	
Chen et al. (2014) [36]	Incidence in acute ward: 2.6%		Incidence in rehabilitation ward (age group wise)
	Incidence in rehabilitation ward:23.2%		< 65 years: 23.4%
			65–75 years: 22.1%
			≥ 75 years: 24.5%
Kim et al. (2014) [37]	Not reported	Not reported	Not reported
Kwon et al. (2014) [38]		Overall prevalence: 91.9%	Prevalence of shoulder pain based on ambulatory mode
			Independent: 93.3%
			Cane: 89.2%
			Wheelchair: 70%
Karaahmet et al. (2014) [39]		Prevalence at admission: 53% Prevalence at discharge: 62%	Prevalence of HSP with other complications
			Neglect: 90%
			Aphasia: 55.6%
			Depression: 65%
			Spasticity: 78.9%
			Sensory disturbance: 40%
			Subluxation: 77.8%
Adey-Wakeling et al. (2015) [3]	Incidence at admission: 10% Incidence at 4 months: 21% Incidence at 12 months: 21% Overall incidence: 29%		Female: 46%
			Medical history
			Previous stroke:12%
			Previous MI: 17%
			Hypertension: 71%
			Diabetes: 28%
			History of shoulder pain: 27%
			Stroke subtype
			Total ischemic: 88%
			Large artery: 14%
			Cardio embolic: 34%
			Lacunar: 9%
			Other/unknown ischemic: 32%
			Hemorrhagic: 9%
			Unknown: 2%
			Left side: 52%
			High NIHSS score (> median): 5%
			Motor arm
			Reduced function: 38%
			No function: 31%
Paolucci et al. (2016) [40]		Overall mean prevalence: 9.41%	
		Acute phase prevalence: 0.63%	
		Sub-acute phase prevalence: 17.27%	
		Chronic phase prevalence:10.34%	

**Table 5:** Shoulder pain and risk factors used in the included studies.

Study	Risk Factors of Shoulder Pain Which Were Assessed	Odd Ratios (OR) [95% Confidence Interval (CI)]
Blennerhassett et al. (2010) [11]	Gender (male)	0.89 (0.36 to 2.16)
	Altered Tone	18.94 (6.46 to 55.51)
	Subluxation	19.34 (5.57 to 65.94)
	Sensory deficits	2.59 (1.08 to 6.17)
	Inattention/neglect	1.53 (0.59 to 3.97)
	Cognitive impairment	1.03 (0.44 to 2.40)
	Impaired communication	1.48 (0.62 to 3.50)
	Type of stroke	0.76 (0.26 to 2.17)
	Hand dominance	0.24 (0.03 to 2.05)
	Previous shoulder problem	2.55 (0.63 to 10.22)
Klit et al. (2011) [32]	Males (vs. females)	0.77 (0.53–1.11)
	Age 65–74 years (vs. < 65 years)	0.57 (0.36–0.90)
	Age > 75 years (vs. < 65 years)	0.65 (0.43–0.99)
	Diabetes (vs. no diabetes)	1.08 (0.65–1.78)
	Depression (vs. no depression)	3.43 (2.25–5.25)
	Infarction (vs. hemorrhage)	0.73 (0.43–1.26)
	Unspecified (vs. hemorrhage)	1.09 (0.57–2.09)
Lindgren et al. (2012) [34]	Left-sided hemiparesis	10.47 (1.92–57.05), *p* = 0.01
	Pain frequency	6.85 (1.46–32.14), *p* = 0.02
	Decreased passive abduction	4.46 (0.99–20.10), *p* = 0.05
	Age	1.05 (0.99–1.12), *p* = 0.07
Kim et al. (2014) [37]	Young age (< 70 years)	3.65 (1.250–10.637), *p* = 0.018
	Male	0.99 (0.370–2.683), *p* = 0.99
	Poor NIHSS item 5 score (≥ 3)	2.96 (1.141–7.665), *p* = 0.026
	Presence of long head of biceps	2.35 (0.897–6.150), *p* = 0.082
	tendon effusion	
	Presence of supraspinatus tendon	4.21 (1.372–12.931), *p* = 0.012
	tendinosis/tear	
Karaahmet et al. (2014) [39]	Neglect	7.20 (0.840–61.4690), *p* = 0.071
	Sensory disturbance	3.26 (0.478–22.301), *p* = 0.228
	Spasticity	1.56 (0.272–9.002), *p* = 0.617
	Immobilization	3.28 (0.527–20.457), *p* = 0.203
	Late rehabilitation	0.52 (0.025–10.658), *p* = 0.669
	Disease duration	1.05 (0.964–1.134), *p* = 0.279
	Baseline FMA (Fugl-Meyer Motor Assessment.)	0.99 (0.905–1.083), *p* = 0.822
	Baseline FAT (Frenchay Arm Test)	
	Baseline FIM (Functional Independence Measure)	0.66 (0.234–1.872), *p* = 0.437
		1.01 (0.970–1.045), *p* = 0.720
Adey-Wakeling et al. (2015) [3]	Mean age (y)	0.96 (0.79–1.17), *p* = 0.690
	Sex: female	1.03 (0.77–1.37), *p* = 0.845
	*Medical history*	
	Previous stroke	0.47 (0.21–1.07), *p* = 0.074
	Previous MI	1.16 (0.53–2.54), *p* = 0.705
	Hypertension	0.96 (0.50–1.85), *p* = 0.907
	Diabetes	1.20 (0.62–2.30), *p* = 0.587
	History of shoulder pain	8.09 (3.16–20.75), *p* = < 0.0001
	*Stroke subtype*	
	Cardioembolic	1.10 (0.60–2.01), *p* = 0.767
	Lacunar	0.85 (0.36–2.04), *p* = 0.719
	Other/unknown ischemic	1.45 (0.78–2.72), *p* = 0.241
	Hemorrhagic	1.22 (0.49–3.01), *p* = 0.670
	Unknown	0.57 (0.09–3.47), *p* = 0.541
	Right Hemiparesis	0.87(0.65–1.17), *p* = 0.350
	High NIHSS score (> median)	1.39 (0.78–2.49), *p* = 0.268
	*Motor arm*	
	Reduced function	1.20 (0.79–1.83), *p* = 0.399
	No function	1.91 (1.20–3.04), *p* = 0.007
